# Deletion of the Mouse Homolog of Human FHR1 (muFHR1) Alleviates Atherosclerosis in ApoE-/- mice

**DOI:** 10.7150/ijms.114990

**Published:** 2026-01-01

**Authors:** Luce Perie, Sarah M. Herr, Tomas Ghebreslassie, Sonia Wulf, Ina Löschmann, Andjela Sekulic, Abdulhadi Suwandi, Anna-Karina B. Maier, Luca Rowlin, Berit Jungnickel, Sascha Schäuble, Gianni Panagiotou, Olaf Strauß, Thorsten Wiech, Peter F. Zipfel, Svante L. H. Zipfel, Christine Skerka

**Affiliations:** 1 Department of Infection Biology, Leibniz Institute for Natural Product Research and Infection Biology, Jena, 07745, Germany.; 2 Institute of Cell Biochemistry, Hannover Medical School, Hannover, 30625 Germany.; 3 Institute of Pathology, University Hospital Hamburg-Eppendorf, 20246 Hamburg, Germany.; 4 Experimental Ophthalmology, Charité - University Medicine Berlin, Corporate Member of Freie Universität, Berlin Institute of Health, Humboldt-University, 10117, Berlin, Germany.; 5 Department of Ophthalmology, Charité - University Medicine Berlin, Corporate Member of Freie Universität, Berlin Institute of Health, Humboldt-University, 10117, Berlin, Germany.; 6 Department of Cell Biology, Institute of Biochemistry and Biophysics, Friedrich-Schiller University, 07745 Jena, Germany; 7 Faculty of Biosciences, Friedrich Schiller University, 07743 Jena, Germany.; 8 Department of Microbiome Dynamics, Leibniz Institute of Natural Product Research and Infection Biology, 07745 Jena, Germany.; 9 Faculty of Medicine, Friedrich Schiller University, 07743 Jena, Germany.; 10 Koania Analytics, 22609 Hamburg, Germany.; 11 Clinic for Heart and Visceral Surgery, University Heart and Vascular Center Hamburg, Medical University Hamburg-Eppendorf, 20246 Hamburg, Germany.

**Keywords:** complement factor H-related protein 1, atherosclerosis, inflammation, cholesterol, oxLDL

## Abstract

Atherosclerosis is the leading cause of heart attack and stroke worldwide. The key characteristic of atherosclerosis is accumulation of LDL cholesterol in artery walls, the subsequent infiltration by monocytes/macrophages, and the development of inflammation. Recently, we reported that plasma protein complement factor H-related 1 (FHR1) binds to the necrotic surfaces of cardiovascular plaques and induces inflammation. Moreover, the concentration of FHR1 is higher, whereas *CFHR1* gene deletion frequency is significantly lower in patients with atherosclerosis in comparison to healthy controls. Here we generated muFHR1-/- (the murine homolog of FHR1) knockout mice and then crossed them with ApoE-/- knockout mice (a model of human hyperlipidemia). Notably, deletion of muFHR1 enhanced lipid conversion in the liver as evidenced by RNAseq analysis. This resulted in normalized cholesterol levels, reduced inflammation and plaque formation in muFHR1-/-ApoE-/- mice. These data suggest that muFHR1 directs uptake of oxLDL by macrophages, and supports foam cell formation, plaque development, and inflammation in dyslipidemic mice. As human FHR1 correlates with non-HDL cholesterol concentrations and inflammation markers in patients with atherosclerosis-associated cardiovascular disease (ACVD) we assume that FHR1 plays a key role in the development of atherosclerosis and subsequent events such as stroke and myocardial infarction.

## Introduction

Inflammation, is an immune response to tissue injury and metabolic stress which, normally, is followed by clearance and healing processes. However, maintenance of an inflammatory state by metabolic deposits in arteries, tissues, or organs can lead to serious inflammatory conditions such as age-related macular degeneration, C3 glomerulopathy, or atherosclerosis-associated cardiovascular disease (ACVD) 1-3. Indeed, ACVD is a leading cause of death and illness in industrialized countries 4-6, highlighting the need for a better understanding of the disease and for the development of new treatments.

Recently, we identified a new player in inflammation mediated by necrotic type surfaces: human dimeric plasma protein factor H-related protein 1 (FHR1). This protein belongs to the human complement factor H protein family, which comprises complement factor H, a splice variant of factor H (factor H-like-1 (FHL-1)), and five FHR proteins (FHR1-FHR-5) 7 - 10. In contrast to factor H and FHL-1, FHR1 does not mediate cleavage of C3b by factor I or accelerate dissociation of the C3 convertase 11. FHR1 binds to complement C3b, C3d, and iC3b, but “prefers” C3d. 11 - 13. Recently, we identified a new function of FHR1 outside the complement system. FHR1 binds to necrotic cell surfaces, such as damaged kidney tissues, in patients with anti-neutrophil cytoplasmic antibody-associated vasculitis, as well as to acellular necrotic cores in the plaques of patients with ACVD 8. After binding, it activates NOD-, LRR, and pyrin domain-containing protein 3 (NLRP3) in monocytes and neutrophils. Upon binding to the G protein-coupled receptor epidermal growth factor-like module-containing mucin-like hormone receptor-like 2 (EMR2), FHR1 induces release of pro-inflammatory cytokines 8. This function is mediated by bound FHR1 but not by soluble FHR1, and serum concentrations correlate with disease progression 14. Having shown that FHR1 binds to atherosclerotic lesions in humans 8, 14, we anticipated that FHR1 exerts inflammatory functions in atherosclerosis. To better understand this potential role of FHR1, particularly its relevance to atherosclerosis, we aimed to investigate the effect of deletion the mouse FHR1 homolog (muFHR1) from a hyperlipidemic mouse model.

Apolipoprotein E (ApoE) in the liver serves as the ligand that clears of all apoB-containing lipoproteins from the blood, except low density lipoprotein (LDL). Thus, knockout of ApoE in mice results in hypercholesterolemia, monocyte proliferation and infiltration into the intima 15-18, oxidative stress, and spontaneous development of atherosclerotic lesions 18. Here, we examined the role of muFHR1 in perpetuating atherosclerosis in muFHR1-/-ApoE-/- double knockout (KO) mice and ApoE-/- mice expressing muFHR1.

## Materials and Methods

### Mice

muFHR1KO (muFHR1-/-) mouse was generated by the MAGEC laboratory (Walter and Eliza Hall Institute of Medical Research, 1G Royal Parade, Vic, 3052, Australia). Briefly, the factor H-related gene 1 (*fhr1*) (NCBI assecion nr NC_000067), was deleted by CRISPR-Cas9 technology in *Mus musculus* strain C57BL/6J chromosome 1 as previously described 20. 20,823 bp of genomic sequence was targeted for deletion (position 139.488536). Both murine and human FHR1 are composed of 5 short consensus repeats, contain the dimerization motif in their first two SCR domains (in contrast to mouse FHRB and FHRC) and show an overall homology between the first SCRs of about 70% (in contrast to FHRB and FHRC with about 40%) 20, 21. In addition, immobilized murine FHR1 binds to necrotic cells and induces IL-1β 8. To avoid confusion and to differentiate between the human and murine FHR1, murine FHR1 is named muFHR1 in the text. ApoEKO mice (B6.129P2-ApoE^tm1Unc/J^ Stock No: 002052) were purchased from Jackson Laboratories. Mice were housed in the FSU animal facility at 24°C. Mice were exposed to a 12-hour light/12-hour dark cycle with free access to normal chow food diet and water. muFHR1+/- and ApoE+/- mice were breed together to generate the muFHR1+/+ApoE+/+ (WT), muFHR1+/+ApoE-/- (ApoE KO), muFHR1-/-ApoE+/+ (muFHR1 KO) and muFHR1-/-;ApoE-/- (muFHR1/ApoE KO) mice. For all the studies genotyped 40-week-old male and female mice were weighed and sacrificed by an overdose of CO_2_ to collect blood, macrophages and tissues. Anesthesia of mice was not used.

### Human serum probes

Serum probes from patients with atherosclerotic-cardiovascular disease (ACVD) were previously described in Irmscher et al 14. Serum probes from healthy control individuals (n=70, mean age 69 ± 11 years, 42 females, 28 males) were obtained from Charité Berlin.

### Cell culture and treatments

Human umbilical vein endothelial (HUVEC) cells were obtained from the American Type Culture Collection (ATCC). HUVEC cells were maintained in growth culture medium (RPMI-1640, Lonza, #12-167F) supplemented with 10% of Fetal Bovine Serum (FBS, ThermoFisher Scientific, # NC0959573) 2 mM Glutamine (Lonza, #BE-17-605EAJ1) and 25 μM Gentamicin (Lonza, #17-518F). Growth medium supplemented with 20% HDL isolated via 12% polyethylene (PEG 6000, Sigma Aldrich)) from normal human serum (NHS) or serum deficient for FHR1/FHR3 was added to the confluent HUVEC cells. After 30 min incubation, cells were treated with 100 ng/ml TNFα for 5h. Cells were then washed with PBS (Lonza, #17-512F) and harvested for RNA analysis.

To isolate peritoneal macrophages, sterile cold PBS was injected according to a procedure as previously described 19. Briefly, after mice euthanasia, PBS was injected in the peritoneal area and then collected and incubated at 4 °C for 30 minutes and then centrifuged. The cell pellet containing the macrophages was resuspended in DMEM (Corning, #10-013-CV), supplemented with 10% FBS, and 1% pen/strep and plated in 12-well tissue culture plates 19. Cells were cultured in DMEM F-12 (Lonza, #BE04-687F/U1) medium supplemented 10% of Fetal Bovine Serum 2 mM Glutamine and 25 μM Gentamicin.

### RNA isolation and RT-PCR analysis

Total RNA was obtained from cultured cells and from tissues with TRIzol (Thermofisher, #15596018). High-Capacity cDNA Reverse Transcription Kit (Applied Biosystems, #4368814) was used to reverse transcribe 1 μg of total RNA into cDNA. RT-PCR analysis was performed using 25 ng of cDNA, 300 nM of primers (listed below) and PowerUp™ SYBR™ Green Master Mix (Applied Biosystems, #A25776) in triplicate, following the manufacturer's instructions. We used the ΔΔCt method for relative mRNA quantification by normalizing each sample to the average change in cycle threshold value of the GAPDH for mouse genes and β-actin for human genes, which were used as control. The following primers were utilized for Q-PCR analysis: — GAPDH Fwd: TGTGTCCGTCGTGGATCTGA; GAPDH Rev: CCTGCTTCACCACCTTCTTGA; muFHR1 Fwd: CATGGTTCTCTACTGCCAAA ; muFHR1 Rev: ATCCTGATCTGTGCAAGTG; TNFα Fwd: CCAGACCCTCACACTCAGATC; TNFα Rev: CACTTGGTGTGCTACGAC; CCL2 Fwd: AGGTCCCTGTCATGCTTCTG; CCL2 Rev: GCTGCTGGTGATCCTCTTGT; IL-6 Fwd: GACAACTTTGGCATTGTGG; IL-6 Rev: ATGCAGGGATGATGTTCTG; IL-10 Fwd: AGCATGGCCCAGAAATCAAG; IL-10 Rev: CGCATCCTGAGGGTCTTCA; LDLR Fwd: ACCCCTCAAGACAGATGGTC; LDLR Rev: CAGCCCAGCTTTGCTCTTAT; HMCGR Fwd: CAACCTCTATATCCGTTTCCAGTCC; HMCGR Rev: TTATGGCAG CAGGCTTCTTGTC; CYP7A1 Fwd: GCTAAGACGCACCTCGTGAT; CYP7A1 Rev: AGGGCTCCTGATCATTTGAA; ABCG5 Fwd: ATTATGTGCATCTTAGGCAGCTC; ABCG5 Rev: CGTAGGAGAAGCAGTCTTGGAA; ACAT2 Fwd: CGATGAGCTAATGGAGGTGC; ACAT2 Rev: GAAGAG GAAGTAGAGGTAGC; CD11c Fwd: GCAGGAGTGTCCAAAGCAAGA; CD11c Rev: CGTGTGCTAGGTCTCTGAAGC; CD68 Fwd: CAAGGTCCAGGGAGGTTGTG; CD68 Rev: CGGAATTTCTG GGATTCAGCTTC; IL-1β Fwd: CTCTCACCTCTCCTACTCACTT; IL-1β Rev: TCAGAATGTGGGAGCGAATG; Arg1 Fwd: CTCCAAGCCAAAGTCCTTAGAG; Arg1 Rev: AGGAGCTGTCATTAGGGACATC; hIL-1β Fwd: CTCTCACCTCTCCTACTCACTT; hIL-1β Rev: TCAGAATGTGGGAGCGAATG; hCXCL10 Fwd: GTGGCATTCAAGGAGTACCTC; hCXCL10 Rev: TGATGGCCTTCGATTCTGGATT; hCCL2 Fwd: AGGTCCCTGTCATGCTTCTG; hCCL2 Rev: GCTGCTGGTGATCCTCTTGT; hβ-actin Fwd: GCTAAGTCCTGCCCTCATTT; hβ-actin Fwd: GTACAGGTCTTTGCGGATGT.

### RNA-sequencing analysis

mRNA from liver samples from 3 animals of each genotype were purified using the total RNA purification kit (Norgen SKU 17200) and professional RNA sequencing (Genewiz) was performed. The distribution of read counts in libraries were examined before and after normalization. The original read counts were TPM (Transcripts Per Kilobase Million) normalized to adjust for gene length, sequencing depth and, sequencing yield between samples. These normalized read counts were used to accurately determine differentially expressed genes. Data quality assessments were performed to detect any samples that are not representative of their group, and thus, may affect the quality of the analysis. The overall similarity among samples were assessed by the Euclidean distance between samples and principal component analysis. This method was used to examine which samples are similar/different to each other. Data of one mouse per condition were subsequently excluded (outlier) as they constantly were out of range. Reads were generated with Illumina NovaSeq in paired-end mode (PE 2x150). Read quality was first assessed with FastQC (v0.12.1). Sequence reads were trimmed to remove possible adapter sequences and nucleotides with poor quality using Trimmomatic (v.0.36). The trimmed reads were mapped to the Mus musculus GRCm38 reference genome available on ENSEMBL using the STAR aligner (v2.5.2b). Unique gene hit counts were calculated by using featureCounts from the Subread package (v.1.5.2). The hit counts were summarized and reported using the gene_id feature in the annotation file. Only unique reads that fell within exon regions were counted. Finally, using DESeq2 (v1.46.0), a comparison of gene expression between groups of samples (wild type, single KO, double KO) was performed. The Wald test was used to generate p-values and log2 of fold changes. Genes with an adjusted p-value < 0.05 and absolute log2 fold change > 0.5 were called as differentially expressed genes. Integration of expression data on KEGG pathway graphs was calculated using pathview (v1.39.0). Enrichment was done using the web application g:Profiler (https://biit.cs.ut.ee/gprofiler/gost) using Benjamini-Hochberg for multiple test correction. KEGG map visualisation was done using pathview (v1.46.0). The R statistical programming language (v4.4.3) was used for RNA sequencing data analysis and visualization unless otherwise noted. All codes are available upon publication at https://github.com/SchSascha/fhr1_apoe_code.

### Enzyme-linked immunosorbent assay (ELISA)

FHR1 binding to ApoB, ApoA1, LDL, ox-ApoB, ox-ApoA1, oxLDL were determined in a sandwich ELISA according the kit manufacturer's instructions and as previously described 14. Briefly, 10μg/ml ApoB, ApoA1, LDL, ox-ApoB, ox-ApoA1 or oxLDL were immobilized onto ELISA plates. After washing with PBS and blocking with 2% BSA-PBS (v/v), 10μg/ml FHR1 was added to the plate. The mixtures were incubated at 37ºC for 1h. Bound FHR1 was detected using monoclonal JHD 7.10.1 antibody, diluted (1:1000) followed by the corresponding anti-mouse secondary antiserum (1:1000). All measurements were performed in triplicate and data for the standard curve were fitted to a logistic plot with the Magellan Data Analysis Software (Tecan).

### Cytokine array

Proteome Profiler Mouse Cytokine Array (R&D Systems, #ARY006) were used following the manufacturer's instructions on mouse serum. The blots were detected using an enhanced chemiluminescence kit on Fusion FX imaging system. Densitometric analysis of the array image files were performed using Image J software. Data analysis and heatmap were generated using Prism 9 software (GraphPad Software).

### Histology

Dissected tissues were fixed in 4% paraformaldehyde (PFA, Sigma, #158127) and embedded in paraffin, according to standard procedures. Tissue sections of hearts of 5 mm thickness derived from 40 weeks old muFHR1-/-ApoE-/-, muFHR1+/+ApoE-/- and WT mice were stained with hematoxylin or Masson-Golner-Elastase was performed following manufacturer's instructions.

### Serum analysis

Serum triglycerides (Sigma, # MAK266), cholesterol (Sigma, # MAK043), Factor H (Abcam, #ab252359), HDL (Crystal Chem., # 79990) LDL (Crystal Chem., # 79980) and C3 (Abcam, #ab157711) levels were measured as per manufacturer's instructions in blood obtained from WT, ApoE-/-, muFHR1-/-, muFHR1-/-ApoE-/- mice. Lipase peroxidation (Abcam, #ab11870) ApoB (R&D Systems, #DAPB00), lipid peroxidation (TBARS Assay, Cayman Chemical #10009055) and FHR1 (Ray Biotech ELH-CFHR1) levels were measured in blood obtained from human donors and ACVD patients. LDL, HDL, triglycerides, glucose and factor V were determined after admission to the hospital. All measurements were performed in triplicate and data for the standard curve were fitted to a logistic plot with the Magellan Data Analysis Software.

### Liver triglyceride analysis

100 mg liver samples were homogenized in phosphate-buffered saline and lipids were extracted using chloroform:methanol (2:1) and 0.1% sulfuric acid. The organic phase was collected, dried and suspended in isopropanol. The levels of triglycerides in liver samples were determined by using the reagents provided in the triglyceride quantification kit (Sigma cat# MAK266), following the manufacturer's instructions, and normalized for liver weights.

### Flow cytometry

Peripheral blood was collected from female wild-type and knockout mice by cardiac puncture into 1.5 mL tubes containing EDTA (final concentration ~2 mM). Red blood cells were lysed using ACK buffer (150 mM NH₄Cl, 10 mM KHCO₃, 0.1 mM EDTA, pH 7.4) for 3-5 min at room temperature, followed by washing with PBS containing 2% FBS and centrifugation at 400 × g for 5 min at 4 °C. Cells were resuspended in FACS buffer (PBS, 2% FBS, 2 mM EDTA) and blocked with anti-mouse CD16/CD32 (BD Biosciences Cat# 553142, RRID:AB_394657) for 10 min on ice. After washing, cells were stained for 30 min at 4 °C in the dark with the following antibodies: CD45-APC/Cy7 (BioLegend Cat# 103116, RRID:AB_312981); CD11b-PE (Thermo Fisher Scientific Cat# 12-0112-82, RRID:AB_2734869); CD11c-FITC (Thermo Fisher Scientific Cat# 11-0114-82, RRID:AB_464940); F4/80-PerCP/Cy5.5 (Thermo Fisher Scientific Cat# 45-4801-82, RRID:AB_914345); Ly6C-APC (BD Biosciences Cat# 560595, RRID:AB 1727554). After washing, cells were resuspended in FACS buffer containing DAPI (1 µg/mL) to exclude dead cells. Samples were acquired on a CytoFLEX flow cytometer (Beckman Coulter) and analyzed using CytExpert software (Beckman Coulter). The Gating strategy was as follows: FSC-A vs SSC-A (exclude debris); FSC-H vs FSC-A (single cells), DAPI⁻ (live cells); CD45⁺ (leukocytes); CD11b⁺ (myeloid cells), then subdivided as: CD11c⁺: dendritic cells; F4/80⁺CD11c⁻: macrophages; Ly6C⁺CD11c⁻: monocytes. Results are expressed as the percentage of each subset within CD45⁺ cells. Absolute counts of CD11b⁺ cells were derived from the volumetric readout of the CytoFLEX cytometer (CytExpert software, Beckman Coulter). CytExpert provided the number of events per µL for each gated population. Absolute counts per mouse were calculated by multiplying events/µL by the starting blood volume (250 µL). Gating was performed on singlet, DAPI⁻, CD45⁺ cells; CD11b⁺; CD11c⁺; F4/80⁺CD11c⁻; and Ly6C⁺CD11c⁻ events were defined from this parent gate.

### Statistical analysis

The results are expressed as mean +/- standard error (SE), unless otherwise noted. Student's t test, or two-way analysis of variance (ANOVA) was used for comparison between groups. One-way ANOVA is indicated in the Figure legend. Pairwise comparisons were performed using Spearman correlation, *p* values <0.05 were considered statistically significant. The statistical analyses were performed using the Prism 9 software.

## Results

### Deficiency of muFHR1 reduces preparedness for inflammation

**Figure 1:**
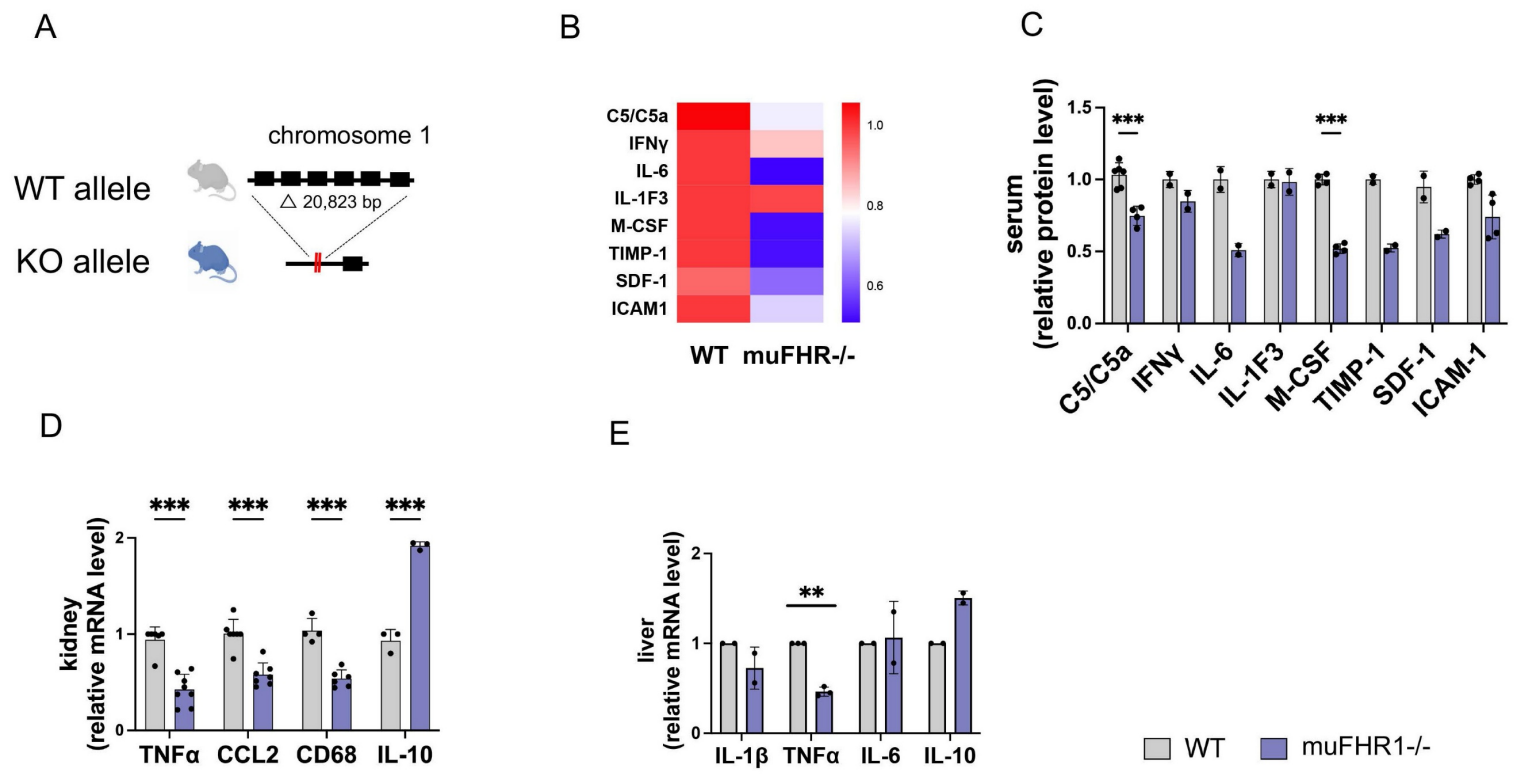
** Characterization of the muFHR1-/- mouse model. (A)** A segment comprising about 20 kbp was deleted from the genome of a black 6 mouse using CRISPR-Cas9 technology; this excised the muFHR1 gene from chromosome 1. (**B**) Heat map showing cytokine levels in the serum of muFHR1-/- and WT mice. Proteins are indicated on the left, p values are shown in C. (**C**) Pro-inflammatory cytokine expression was lower in muFHR1-/- mice than in WT mice (***p < 0.001, p** < 0.01 two-way ANOVA with the first gene expression in WT set as 1, n = 2-4). (**D**) Cytokine mRNA profiles in kidney and liver cells (**E**) confirmed reduced expression of pro-inflammatory gene in muFHR1-/- mice (***p < 0.001, **p<0.01, two-way ANOVA, with the first gene expression in WT set as 1, n = 2-8).

Having shown previously that FHR1 promotes inflammation by inducing monocytes/macrophages and granulocytic neutrophils *in vitro,* and that FHR1 is associated with inflammation in ACVD, we wanted to identify the function of FHR1* in vivo*. For this purpose, we generated muFHR1 KO mice (muFHR1-/-) using CRISPR-Cas9 technology (Fig. 1A) as previously described 20 and then crossed them with ApoE-/- mice. Initial phenotypical analysis of 40-week-old male and female muFHR1**-/-** mice fed a normal chow diet demonstrated that body and tissue weights were comparable with those of WT mice supplementary Fig. 1A and 1B). muFHR1 is a complement protein; therefore, we also evaluated whether expression of complement component C3 and factor H were altered in muFHR1**-/-** mice. There was no significant difference in C3 and factor H levels between WT and muFHR1**-/-** mice (supplementary Fig. 1C and 1D). Previous data show that FHR1 induces the NLRP3 inflammasome in monocytes; therefore, we analyzed expression of inflammatory molecules in muFHR1**-/-** mice at the RNA and protein levels. To exclude different numbers of monocytes in muFHR1**-/-** mice compared to WT mice, the percentage of monocytes was determined in the CD45^+^ leucocytes of the mice. The mice showed no significant difference in their leucocyte populations and subgroups of CD45^+^ cells, especially in the subgroup of CD45^+^ cells being Ly6C^+^ and CD11c^-^ which represents predominantly monocytes (Supplementary Fig. 1E to H). There was also no significant difference in the absolute number of cells measured. (Supplementary Fig. 1I to 1L). Cytokine array analysis of muFHR1-/- mouse serum revealed that expression levels of C5a and M-CSF were significantly reduced in muFHR1-/- than in WT mice (Fig. 1B and C). A reduced inflammation level was confirmed by mRNA analyses of kidneys from muFHR1-/- mice, which showed that expression of TNFα, CCL2, and CD68 mRNA (Fig. 1D) was significantly lower (p < 0.001, 2-way ANOVA) than in WT mice expressing muFHR1. Expression in liver cells revealed significant reduction of TNFα (Fig. 1E) gene expression. By contrast, expression of the anti-inflammatory marker IL-10 increased (Fig. 1E). Taken together, these data show that muFHR1**-/-** mice are vital, and that muFHR1 affects expression of inflammatory genes.

### Deletion of muFHR1 from ApoE-/- mice attenuates inflammatory responses

**Figure 2:**
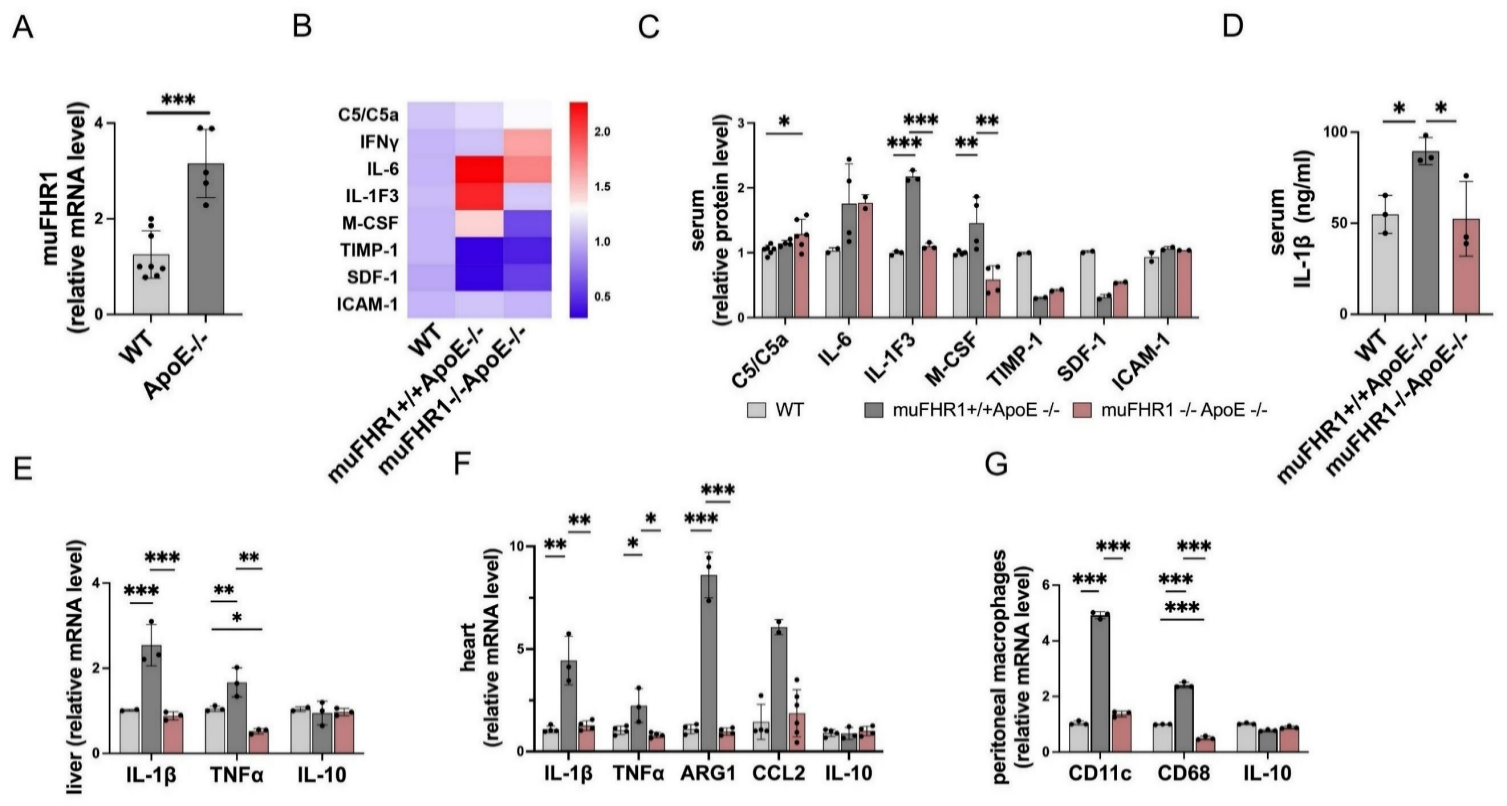
** Expression of pro-inflammatory proteins in muFHR1-/-ApoE-/- and muFHR1+/+ApoE-/- mice.** (**A**) muFHR1 protein levels were significantly higher in muFHR1+/+ApoE-/- mice than in WT mice (***p<0.001, unpaired Student's t-test, n=5-8). (**B**) Heat map showing cytokine levels in serum from muFHR1-/-ApoE-/- mice and muFHR1+/+ApoE-/- mice. Proteins are indicated on the left, p values are shown in C. (**C**) Pro-inflammatory cytokine levels are significantly lower in muFHR1-/-ApoE-/- mice than in muFHR1+/+ApoE-/- or WT mice, n=2-6. (**D**) IL-1β protein levels in serum from muFHR1-/-ApoE-/- mice are significantly lower than those in muFHR1+/+ApoE-/- mice (***p<0.001, p**<0.01, *p<0.05, two-way ANOVA, n=3). (**E**) Expression of mRNA encoding IL-1β, TNFα and IL-10 in muFHR1-/-ApoE-/- mice compared to muFHR1+/+ApoE-/- mice, n=3. (**F**) Expression of pro-inflammatory genes in heart cells from muFHR1-/-ApoE-/- mice is significantly lower than that in cells from muFHR1+/+ApoE-/- mice, n=3-6. (**G**) Peritoneal macrophages from muFHR1-/-ApoE-/- mice express significantly lower levels of differentiation markers CD11c and CD86 than those from muFHR1+/+ApoE-/- mice, n=3. Data in **C**, **E-G**: ***p<0.001, p**<0.01, *p<0.05, two-way ANOVA with the first gene expression in WT set as 1.

Because FHR1 is highly expressed in ACVD patients (15, we next asked how muFHR1 effects inflammation in ApoE**-/-** mice. First, we measured liver gene expression of muFHR1 in ApoE-/- mice, and found that expression was much higher in 40-weeks-old ApoE-/- mice than in similar aged WT mice (about 3-fold, p<0.001, student's t-test) (Fig.2A). We crossed muFHR1-/- mice with ApoE-/- mice to generate muFHR1-/-ApoE-/- double knock out (KO) mice and examined changes in the inflammatory response in muFHR1-/-ApoE-/- mice using again a cytokine array. Compared with ApoE-/- mice, which express muFHR1, IL1-F3, and the macrophage colony-stimulating factor M-CSF, were significantly lower in the serum of muFHR1-/-ApoE-/- mice compared to ApoE-/- mice (*p < 0.05, **p < 0.01, ***p < 0.001, 2-way ANOVA) (Fig. 2B and 2C). No differences were seen in C5/C5a, IL6, Timp-1, SDF-1 and ICAM-1 expression. Separate measurement of serum IL-1β revealed significantly lower levels (*p < 0.05, 2-way ANOVA) in the double KO mice than in ApoE-/- mice (Fig. 2D). Analysis of mRNA levels in liver and heart tissues from double KO mice revealed the same results. Expression of IL-1β and TNFα in the liver and heart of double KO mice was markedly lower than in that of ApoE-/- mice, and were similar to WT mice (Fig. 2E). In addition, high gene expression of Arg1 and chemokine CCL2 in heart tissue of ApoE-/- were normalized in muFHR1-/-ApoE-/- mice (Fig. 2F). Furthermore, immuno-phenotyping of peritoneal macrophages revealed a reduction in the pro-inflammatory macrophage (M1) markers in muFHR1-/-ApoE-/- mice, as demonstrated by low expression levels of CD68 and CD11c (Fig. 2G). Altogether these data demonstrate that loss of muFHR1 from this hyperlipidemic mouse model decreases inflammation, reduces M1 polarization, and dampens the inflammatory response.

### Deleting muFHR1 reduces accumulation of lipids and cholesterol

**Figure 3:**
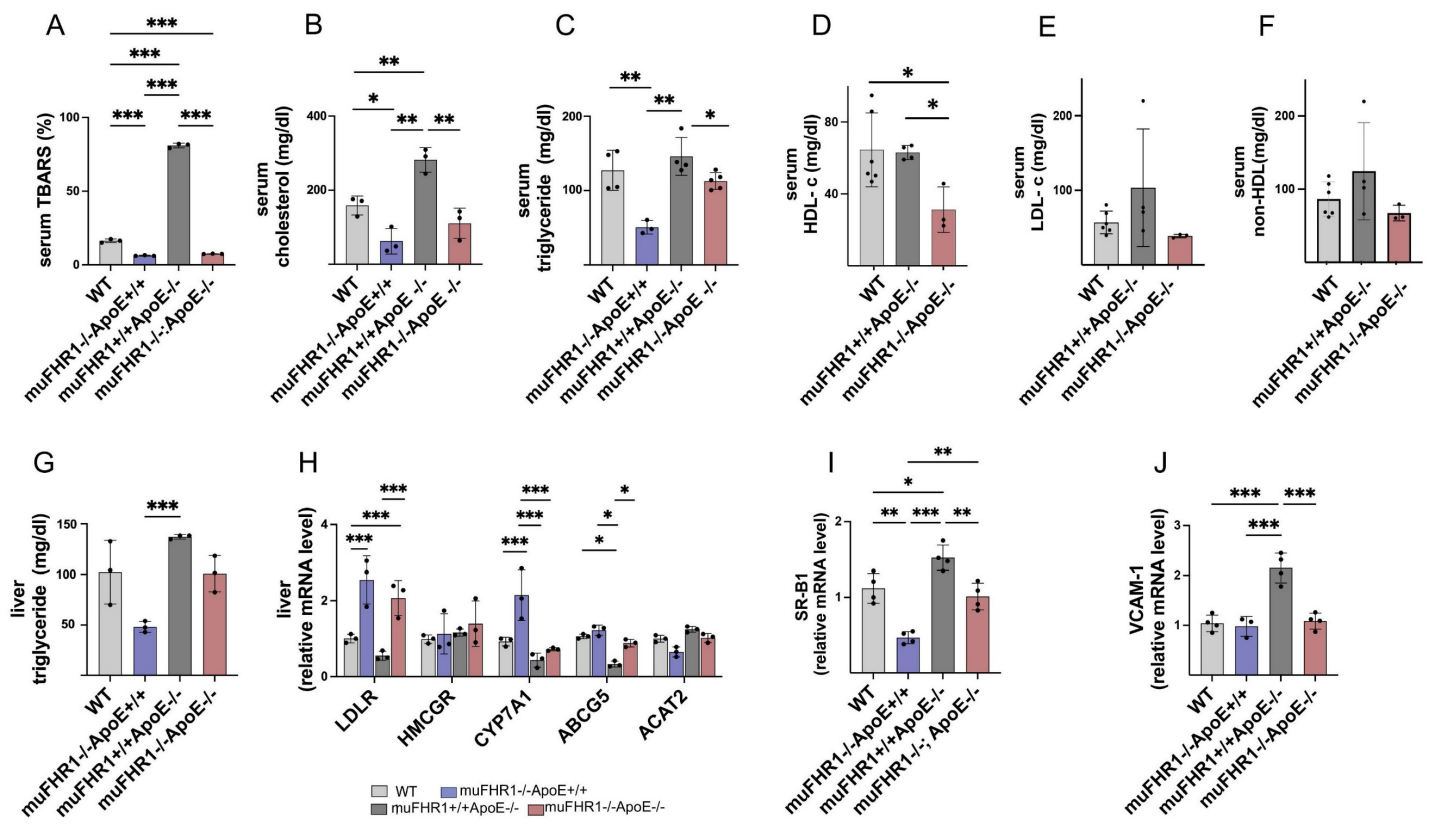
** muFHR1 deficiency normalizes lipid accumulation and oxidation in muFHR1+/+ApoE-/- mice.** (**A**) High levels of lipid oxidation in the serum of muFHR1+/+APOE-/- mice return to normal upon deletion of muFHR1 (n = 3) (**B**) High levels of serum cholesterol and (**C**) triglyceride in the serum of muFHR1+/+APOE-/- mice return to normal upon deletion of muFHR1 (n = 4). (**D**) Serum HDL levels are significantly reduced in muFHR1-/-ApoE-/- mice compared to WT and muFHR1+/+ApoE-/- mice (Student's t-test, n = 3-6). (**E**) Serum LDL and (**F**) serum non HDL are normalized in muFHR1-/-APOE-/- mice. (**G**) Elevated triglycerides seen in the liver of muFHR1+/+ApoE-/- mice are reduced markedly in muFHR1-/-APOE-/- mice (n = 3). (**H**) LDLR gene expression in muFHR1-/- mice and double KO muFHR1-/-APOE-/- mice is significantly higher than that in muFHR1+/+ApoE-/- and WT mice. CYP7A1 is highly expressed in muFHR1-/-ApoE+/+ mice, but not in WT, muFHR1-/-ApoE-/-, or muFHR1+/+ApoE-/- mice. HMCGR, ABCG5, and ACAT2 are expressed at low levels in all mouse strains (n=3). (**I**) Expression of VCAM-1 and SR-B1 in muFHR1-/-ApoE-/- mice is significantly lower than that in muFHR1+/+ApoE-/- mice (n = 3-4). Data in **A-D**: ***p < 0.001, p** < 0.01, *p < 0.05, two-way ANOVA. Data in **E-G**: ***p < 0.001, p**< 0.01, *p < 0.05, two-way ANOVA with the first gene expression in WT set as 1.

Atherosclerosis is an inflammatory disorder characterized by accumulation of LDL cholesterol in artery walls, followed by infiltration by monocytes/macrophages and lipid peroxidation. To find out how muFHR1 modulates inflammation in ApoE-/- mice, we measured the level of lipid oxidation in the serum of all four animal models using the TBARS assay kit. As expected, ApoE-/- mice had high TBARS levels (78.8 ± 2.9 %). By contrast, levels were much lower (5.8 times) in muFHR1-/-ApoE-/- mice (13.5 ± 2 %) (Fig.3A); indeed, they were significantly lower than those in WT mice (17.8 ± 1.7 %) (***p < 0.001, 2-way ANOVA). TBARS level was significantly lower (***p < 0.001, 2-way ANOVA) in muFHR1-/- mice (12.7 ± 1.7 %) compared to WT mice. These results demonstrate that deleting muFHR1 from ApoE mice normalizes lipid peroxidation levels, highlighting potential involvement of muFHR1 in lipid oxidation and atherosclerosis.

To further evaluate the role of muFHR1 in lipid metabolism, we measured the levels of cholesterol and triglycerides in the serum of WT, muFHR1-/-, ApoE-/-, and muFHR1-/-ApoE-/- mice. We noted a significant reduction (*p<0.05, **p<0.01, 2-way ANOVA) in cholesterol and triglycerides in serum from both muFHR1-/- and muFHR1-/-ApoE-/- mice compared with ApoE-/- mice (Fig. 3B and 3C). For more specificity HDL and LDL serum levels were measured, showing similar levels of HDL in muFHR1+/+ApoE-/- compared to WT mice but significantly reduced levels in muFHR1-/-ApoE-/- mice (Fig. 3D). LDL concentrations reflect the cholesterol levels shown in Fig. 3B, with normalized levels in muFHR1-/-ApoE-/- compared to muFHR1+/+ApoE-/- mice (Fig. 3E). Non-HDL serum levels revealed similar results with elevated levels in muFHR1+/+ApoE-/- mice which normalized in muFHR1-/-ApoE-/- animals (Fig. 3F). To confirm reduction of serum triglyceride results, we also measured triglyceride levels in the liver. Again, levels in muFHR1-/- mice were lower than those in ApoE-/- mice. Notably, liver triglyceride levels in muFHR1-/-ApoE-/- mice were similar to those in WT mice (Fig. 3G). In summary, serum lipid oxidation, cholesterol, triglycerides, LDL as well as non-HDL levels in muFHR1-/-ApoE-/- mice were normalized compared to muFHR1+/+ApoE-/- mice. These results suggest that muFHR1 regulates the lipid composition of blood.

### muFHR1 mediates cholesterol metabolism in the liver

Altered cholesterol efflux in the liver is a hallmark of atherosclerosis 22. Therefore, to examine the effect of muFHR1 on cholesterol uptake in the liver we measured expression of genes involved in the cholesterol metabolic pathway by RNA expression levels in the liver. These include the LDL receptor (LDLR), which is involved in LDL uptake; 3-hydroxy-3-methyl-glutaryl-coenzyme A reductase (HMCGR), which catalyzes synthesis of cholesterol and other lipids; cholesterol 7-alpha-monooxygenase or cytochrome P450 7A1 (CYP7A1), which is involved in synthesis of bile acids from cholesterol; ATP-binding cassette sub-family G member 5 (ABCG5), which promotes biliary excretion of sterols; and Acetyl-Coenzyme A acetyltransferase 2 (ACAT2), which mediates cholesterol esterification and generation of VLDL 23 - 27. QPCR analysis of liver cells revealed that muFHR1 deficiency in ApoE-/- mice leads to a marked increase in expression of the LDLR receptor (by 1- to 2-fold compared with WT), suggesting increased uptake of cholesterol by the liver (Fig. 3H). In addition, expression of genes encoding CYP7A1 (but not HMCGR and ACAT2) was significantly higher in muFHR1-deficient animals than in WT animals (***p < 0.001, 2way ANOVA), indicating that increased cholesterol uptake by the LDLR increases bile formation and reduces VLDL production. AbCG5 expression was similar to WT animals but low in ApoE-/- mice (Fig. 3H). The effect on HDL was explained when we examined the blood monocyte recruitment receptors SR-B1 and VCAM-1 28, as expression of these receptors was reduced significantly in muFHR1-/-ApoE-/- mice compared with ApoE-/- mice (Fig. 3I, 3J).

**Figure 4:**
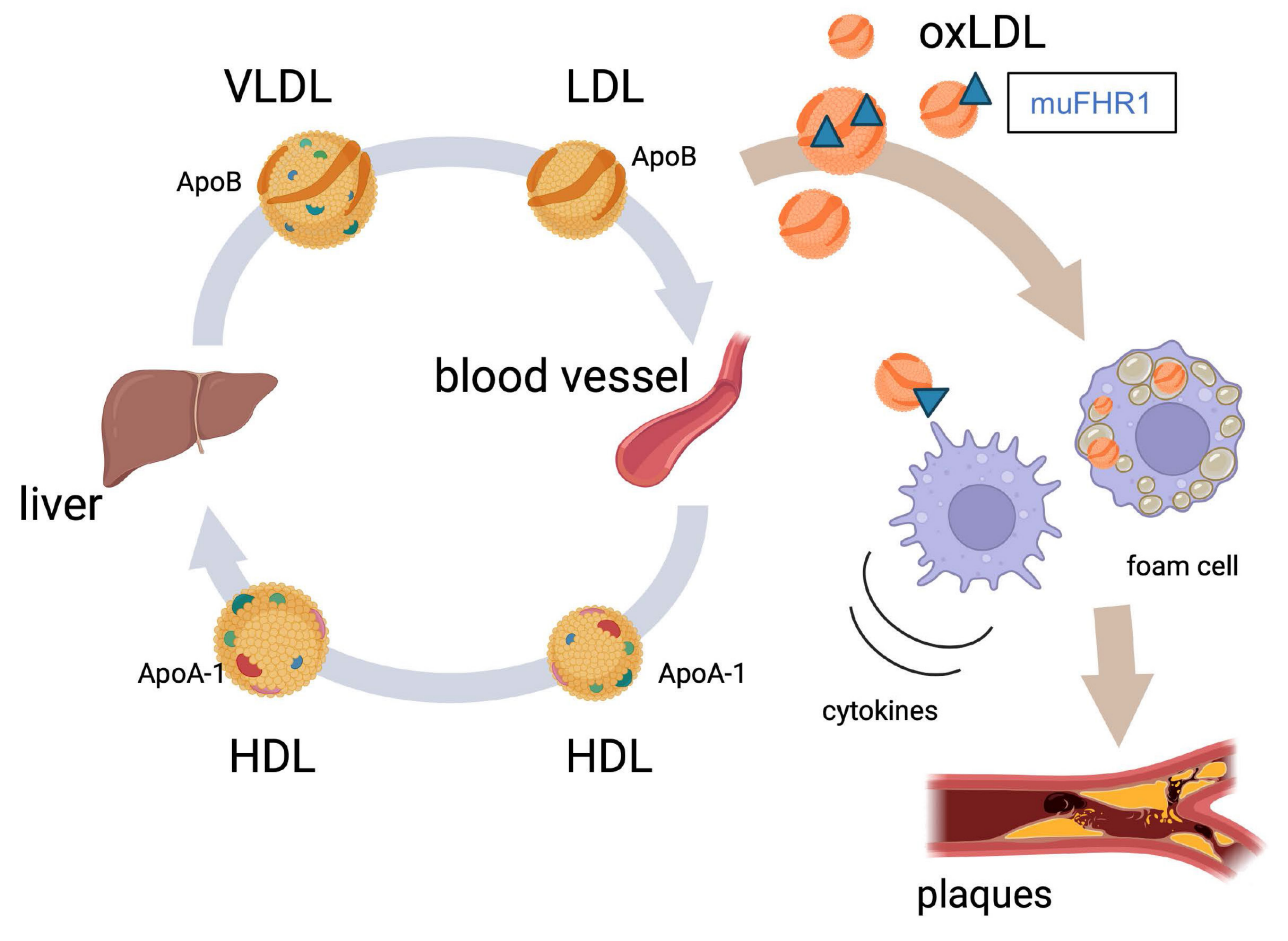
Model illustrating the role of FHR1 (muFHR1) in atherosclerosis: muFHR1 binds to accumulating oxLDL (oxApoB100) leading to activation of monocytes and macrophages to secrete pro-inflammatory cytokines.

These data suggest that muFHR1 deficiency in atherosclerosis improves clearance of LDL-cholesterol, thereby reducing lipid oxidation and inflammation. The possible role of FHR1 is summarized in a model (Fig.4). These results may explain the normal LDL levels in muFHR1-/-ApoE-/- mice, as well as the protective effects of muFHR1 deficiency against cardiovascular disease in ApoE-/- mice.

### muFHR1 deficiency enhances lipid catabolism in ApoE-/- mice

**Figure 5:**
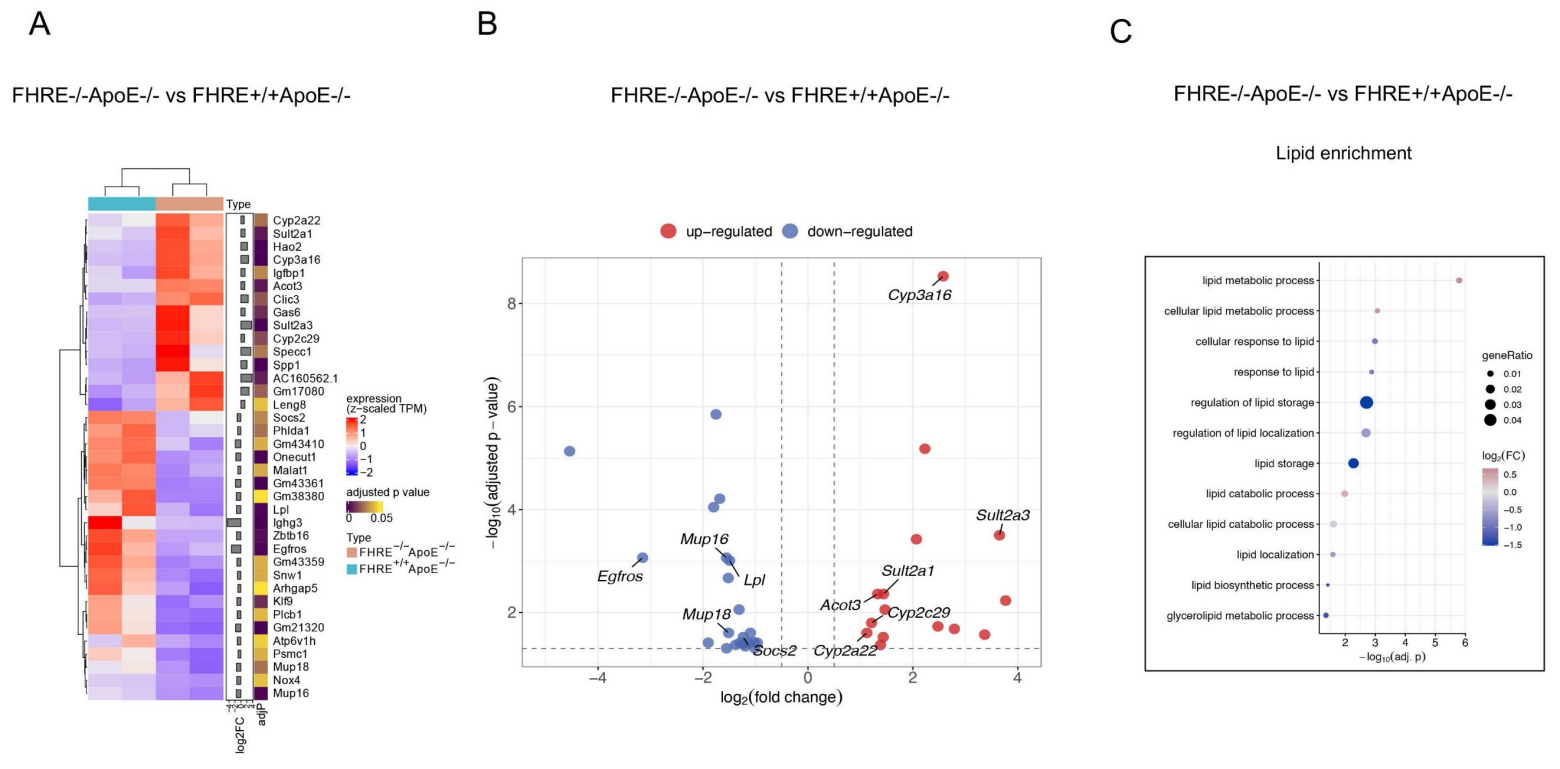
** Transcriptional changes in the liver induced by muFHR1 deficiency in ApoE-/-mice.** (**A**) Heatmap of DEGs in muFHR1-/-ApoE-/- mice compared to muFHR1+/+ApoE-/- mice (n=2 samples per group) showing the effects of muFHR1 deficiency. Log2 of fold changes (log2FC) and multiple test corrected p values by FDR are indicated. (**B**) Volcano plot of significant DEGs (P < 0.05) in the liver of the comparisons muFHR1-/-ApoE-/- versus muFHR1+/+ApoE-/- mice, highlighting the increased expression of the cytochrome P450 oxidreductases Cyp3a16, Cyp2c29 and Cyp2a22 and the sulfotransferase genes Sult 2a1 and Sult2a3. (C) Lipid enrichment analysis confirmed the association of the induced genes with lipid metabolism and lipid storage.

In order to ascertain the reason behind the low cholesterol levels observed in muFHR1-deficient mice, the RNA liver expression profiles of the different mouse strains were identified, given that lipids are metabolized primarily in the liver. For this reason, total RNA was extracted from the livers of 40-week-old ApoE-/- and muFHR1-/-ApoE-/- mice, followed by RNA sequencing (RNAseq) analysis. The data demonstrated that in muFHR1-/-ApoE-/- mice, genes were subject to differential regulation in comparison to ApoE-/- mice (Figure 5A). As anticipated, numerous upregulated genes were linked to lipid metabolism (Figures 5B and 5C), including those involved in lipid storage and conversion. The increased gene expression of members of the P450 oxidoreductase family, which are involved in the conversion of lipid acids supplementary Figure 3 and supplementary Figure 4, may be responsible for the reduction in serum cholesterol levels observed in muFHR1-/-ApoE-/- mice compared to ApoE-/- mice. Additionally, the sulfotransferases Sult2a1 and Sult2a3 exhibited increased expression, indicating enhanced sulphation of bile acids and an increase in water solubility, which consequently resulted in enhanced renal secretion. This represents a crucial pathway for the detoxification and excretion of bile acids. 29, 30. Conversely, lipoprotein lipase (LPL) expression was reduced in muFHR1-/-ApoE-/- mice relative to ApoE-/- mice. Lipoprotein lipase (LPL) plays a crucial role in the metabolism of very low-density lipoprotein (VLDL) particles by facilitating their conversion into intermediate-density lipoproteins (IDL) and low-density lipoproteins (LDL) 6.

### muFHR1 deficient ApoE-/- mice show less atherosclerotic plaques

**Figure 6:**
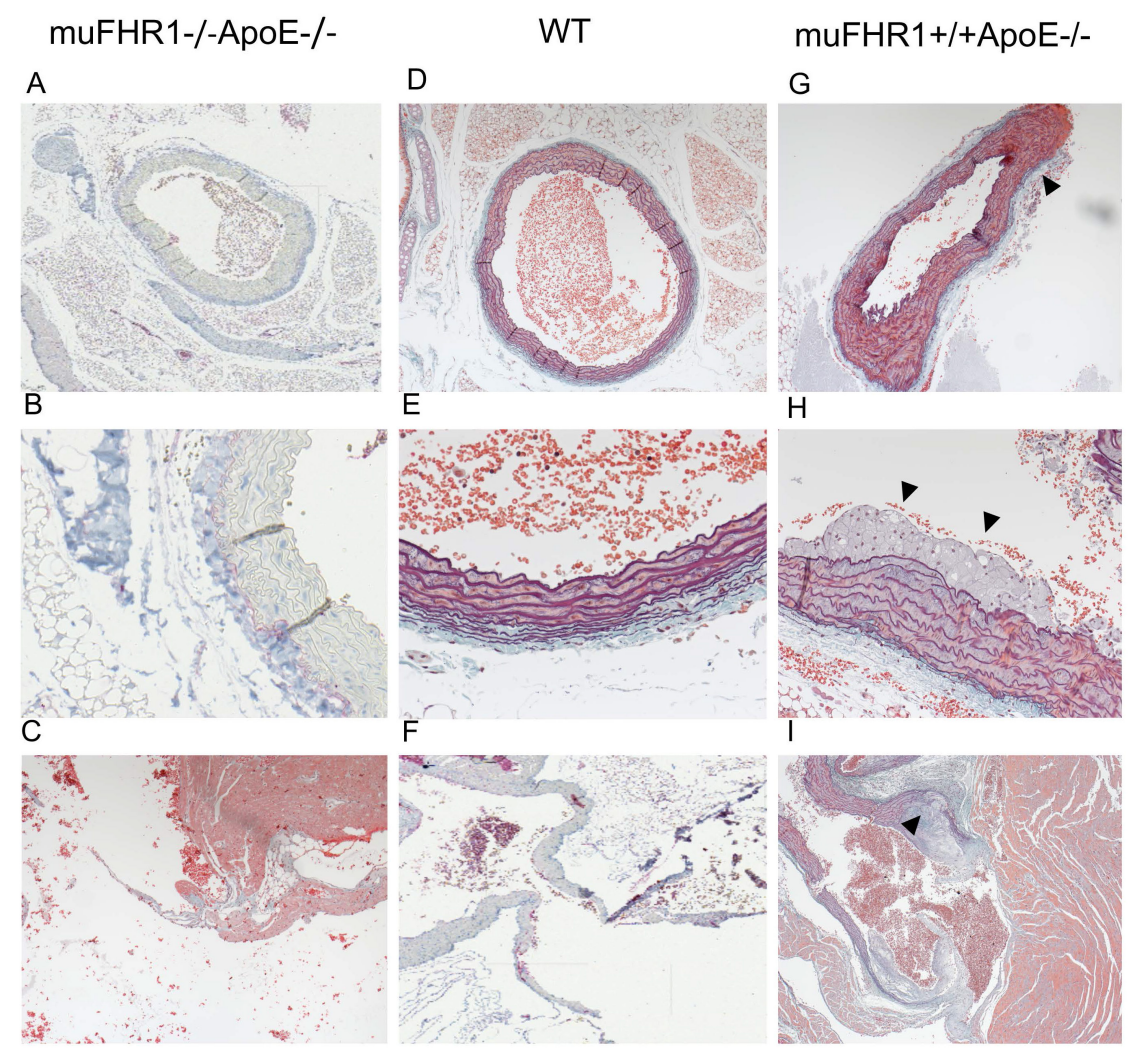
** The muFHR1-/- ApoE-/- mouse is free of atherosclerotic plaques.** The paraffin-embedded sections of hearts of 40-week-old muFHR1-/-ApoE-/- (**A-C**), WT (**D-F**) and muFHR1+/+ApoE-/- (**G-I**) mice stained with hematoxylin or Masson-Goldner-Elastica revealed (**A-C**) that the aorta and valves in muFHR1-/-ApoE-/- mice show normal architecture and are free of deposits (**D-F**) comparable to WT mice. In contrast, (**G**) muFHR1+/+ApoE-/- mice display a slight enlargement of the aortic adventitia (triangle) (magnification 10x), (**H**) multilayered intimal foam cell deposits (triangles) (magnification x 26) (see also supplementary Figure 2, and (**I**) enlarged aortic valves with atherosclerotic plaques (triangle) containing cholesterol crystals (magnification 26). Panel D, E and I illustrate the exclusive use of hematoxylin staining.

Given the normalized LDL serum levels in muFHR1-/-ApoE-/- mice we sought to determine whether these mice develop atherosclerotic plaques by histochemistry. Hearts from 40-week- old muFHR1-/-ApoE-/-, ApoE-/- and WT animals fed normal mouse chow were embedded into paraffin and sections stained with hematoxylin or Masson-Goldner-Elastica. Examination of aortic sections revealed no abnormalities in muFHR1-/-ApoE-/- mice (n=3), no foci of inflammation and the luminal surface of the aorta was smooth with orderly arranged smooth muscle cells (Figure 6A-C). No evidence of either plaque formation or inflammatory activity was detected in several screened sections of muFHR1-/-ApoE-/- mice (>10 of n=3). The overall architecture was comparable to WT mice (Figure 6D-F). In contrast, examination of sections of ApoE-/- mice revealed slightly enlarged aortic walls (Figure 6G) and intimal multilayered foam cell deposits in the aortic outflow (Figure 6H, supplementary Figure 2A). Multilayered foam cell deposits were found adjacent to valve attachment sites in the form of continuous deposits containing cholesterol crystals (Figure 6I, supplementary Figure 2B).

### Human FHR1 serum concentrations correlate with atherosclerosis markers in ACVD patients

**Figure 7:**
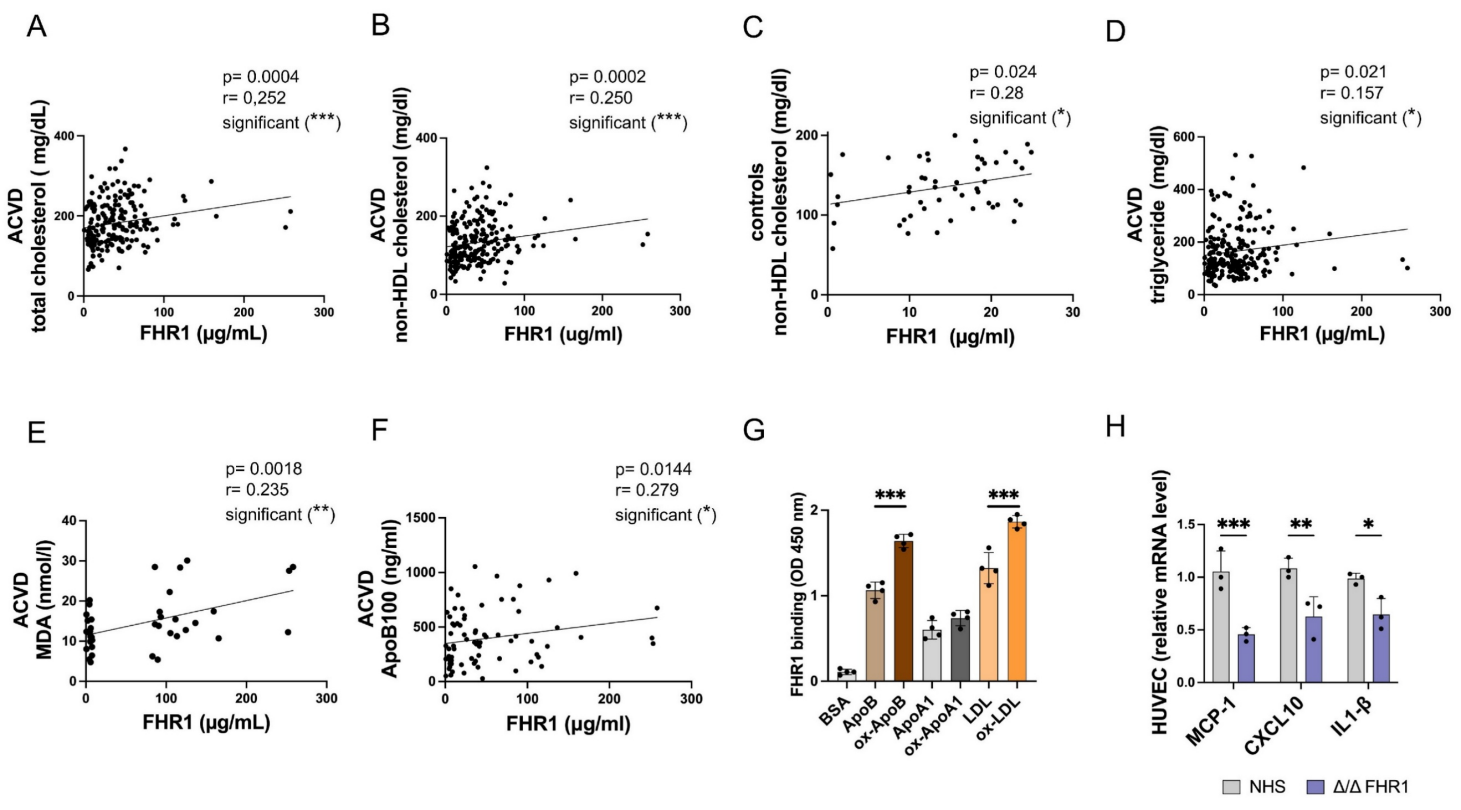
** Serum FHR1 concentrations correlate with non-HDL cholesterol levels in ACVD patients.** (**A**) serum FHR1 concentrations in ACVD patients correlate with total cholesterol (n=192) and (**B**) non-HDL cholesterol (n=211). (**C**) Although FHR1 concentrations are substantially lower in serum of aged matched healthy control individuals compared to ACVD patients, they correlate with non-HDL cholesterol levels (n=50). (**D**) In addition, FHR1 serum concentrations of ACVD patients correlate with serum triglycerides (n=213), (**E**) lipid peroxidation levels (n=33), and (**F**) serum concentrations of ApoB100 (n=76). (**G**) FHR1 binding to ApoB100 and LDL is significantly enhanced upon oxidization. No enhancement was detected upon binding to oxidized ApoA1 (***p<0.001, one-way ANOVA, n=3-4). (**H**) Expression of pro-inflammatory cytokines MCP-1, CXCL10, and IL-1β in HUVEC cells is decreased upon incubation with HDL isolated from normal human sera not containing FHR1 (***p<0.001, p**<0.01, *p<0.05, two-way ANOVA, n=3). A-E: Spearman correlations.

Previously, we showed that high levels of FHR1 correlate with LDL but not HDL in atherosclerotic patients 15, suggesting that binding of FHR1 to both oxLDL and monocytes plays a role in formation of atherosclerotic plaques. Given the data presented above, we hypothesized that by binding of FHR1 to oxLDL FHR1 disrupts reverse cholesterol transport to the liver and directs uptake of oxLDL by macrophages within plaques, leading to inflammation. To test this, we measured total and non-HDL cholesterol as well as triglycerides in serum samples from our previously described ACVD cohort 14, 15. FHR1 concentrations correlated significantly with total cholesterol (***p = 0.0004, Spearman correlation) (Fig. 7A) and especially non-HDL cholesterol in patients (***p = 0.0002, Spearman correlation) (Fig. 7B), but not with glucose levels supplementary Fig. 5A). Measuring FHR1 and non-HDL cholesterol levels in a healthy control cohort revealed lower FHR1 concentrations, but already a weak significant correlation of FHR1 with non-HDL values (Fig. 7C). The data confirm a close relationship between FHR1 and cholesterol levels in humans. In ACVD patients FHR1 serum concentrations correlated significantly also with triglycerides (Fig. 7D) and strongly with oxidation levels measured by malondialdehyde (MDA) analysis (Fig. 7E) as well as ApoB100 (Fig. 7F). Moreover, FHR1 concentrations in ACVD patients correlate with coagulation factor V (supplementary Figure 5B) reflecting the strong association of FHR1 with atherosclerotic markers.

Apolipoprotein ApoB100 is required for assembly of VLDL in the liver, and also serves as the primary structural and functional ligand of LDL 6. Because FHR1 binds to oxLDL *in vitro* 8, 14, 31, and factor H and FHR4 bind to apolipoproteins 32, 33, we next measured binding of FHR1 to ApoB100, ApoA1 (HDL ligand), and LDL (as well as their oxidized forms) in an ELISA. When FHR1 binding to ApoB100 was set to 1, binding increased significantly (***p< 0.001, 1-way ANOVA) upon oxidization of ApoB100 (ox-ApoB100) (Fig. 7G). The same increase in FHR1 binding activity was observed for LDL versus oxLDL. By contrast, FHR1 did not bind strongly to oxidized apolipoprotein ApoA1, the structural protein of HDL. These data confirm that FHR1 binds preferentially to ox-ApoB100 and oxLDL rather than to the non-oxidized forms. oxLDL activates macrophages within lesions in inflammatory plaques 6.

Previous studies have shown that HDL reduces inflammation 34. Based on our results showing that FHR1 modulates the LDL-cholesterol serum concentrations, we examined the effect of FHR1 on the anti-inflammatory activity of HDL. HUVEC cells were treated with TNFα and then incubated with HDL isolated from human FHR1-/- serum. We then examined expression of pro-inflammatory genes. Levels of mRNA encoding pro-inflammatory markers CXCL10 and CCL2 fell significantly when HUVEC cells were incubated with HDL isolated from FHR1-deficient serum (Fig. 7H).

## Discussion

Atherosclerosis is the underlying cause of sudden heart attack and stroke 5, 6. Here, we identified a key protein, FHR1, that may play a pivotal role in the development of atherosclerosis. FHR1 binds to oxidized LDL and activates monocytes/macrophages 8, 14. Therefore, we asked whether FHR1 bound to surfaces such as atherosclerotic plaques affects pro-inflammation and progression of the disease. Deleting the human FHR1 homolog muFHR1 from hyperlipidemic ApoE-/- mice normalizes plasma LDL cholesterol levels, reduces atherosclerotic lesions and inflammation in these mice. Moreover, elevated FHR1 levels in serum from ACVD patients correlates with oxLDL and serum oxidation levels, with non-HDL cholesterol and coagulation factor V levels, but not with HDL or glucose levels. Thus, the data suggest that FHR1 fuels inflammation in atherosclerosis via binding to increased presence of oxLDL.

muFHR1-/- mice are phenotypically similar to WT mice; both have similar body weight and show similar expression of complement factors such as factor H or C3. This agrees with a recent report describing muFHR1-/- mice 35. Previously, we showed that FHR1 (and muFHR1) binds to necrotic monocytes and neutrophils, and activates expression of pro-inflammatory genes 8. Thus, it is not surprising that 40-week-old muFHR1-/- mice have significantly lower serum levels of factors involved in inflammation (e.g., C5 (C5a) and monocyte colony stimulating factor (M-CSF)). Also, levels of the metallopeptidase inhibitor TIMP-1 are significantly lower than in WT mice, as levels of the intracellular migration protein ICAM1 (which promotes transmigration of immune cells) and stromal cell-derived factor 1 (SDF-1), a C-X-C motif chemokine (CXCL12) that strongly attracts lymphocytes 36, were reduced, however without statistical significance. Kidney and liver cells show lower expression of mRNA encoding inflammatory cytokines and higher expression of IL-10 mRNA, than WT mice, confirming the more anti-inflammatory background of muFHR1-/- mice.

However, the pro-inflammatory function of muFHR1 becomes much clearer when the hyperlipidemic mouse model (ApoE-/-) expressing muFHR1 is compared with the model not expressing muFHR1. Serum from muFHR1-/-ApoE-/- mice contained significantly less inflammatory cytokine IL-1F3 and monocyte differentiation and proliferation stimulating factor M-CSF than serum from ApoE-/- mice. In addition, serum IL-1β concentration normalized. Deletion of muFHR1 from ApoE-/- mice reduces expression of inflammatory genes encoding IL-1β and TNFα in the liver and heart, and normalizes expression of arginase 1 (Arg1) for the biosynthesis of nitric oxide from arginine and monocyte chemotactic protein 1 CCL2. Overall, muFHR1 deficiency in APOE-/- mice normalizes pro-inflammatory gene expression and inhibits expression of differentiation markers CD11c and CD68 in peritoneal macrophages. The *in vivo* data suggest that presence of muFHR1 supports inflammation in atherosclerosis.

Deleting the lipid transporter ApoE from mice increases triglycerides and total cholesterol, thereby inducing hypercholesterolemia, spontaneous development of atherosclerotic lesions, and inflammation 16 - 18. Measurement of serum lipid levels in ApoE-/- mice confirm elevated serum levels of triglycerides and cholesterol. Notably, deletion of muFHR1 from ApoE-/- mice normalizes triglycerides in serum and liver cells, total cholesterol concentrations, as well as LDL and non-HDL serum concentrations. The underlying mechanisms by which muFHR1 modulates cholesterol and triglyceride levels required further analysis.

ApoE-/- mice show high levels of serum cholesterol as well as oxidation levels and develop atherosclerotic plaques. In addition, muFHR1 is significantly elevated in serum of hyperlipidemic ApoE-/- mice. In contrast, muFHR1-/-ApoE-/- mice have very low oxLDL cholesterol concentrations and LDL levels are substantially lower in single KO muFHR1-/- and double KO muFHR1-/-ApoE-/- mice compared to ApoE-/- mice; suggesting that LDL is likely to be transported to the liver. RNAseq analysis of the liver confirmed this hypothesis as data revealed a significant increase in the expression of cholesterol and bile acid metabolism-related genes. Thus, we suggest that LDL cholesterol in muFHR1-/-ApoE-/- mice is cleared by increased uptake via upregulated LDL receptors, followed by conversion to bile acids, as indicated by increased expression of Cyp7A1, Cyp3a16 and Cyp2a22 and of sulfotransferases for secretion of bile acids. Indeed, LDLR, whose function is mainly related to LDL/VLDL uptake and subsequent conversion 37 is significantly upregulated in liver cells of single KO muFHR1-/- and double KO muFHR1-/-ApoE-/- mice. In contrast to LDLR, SR-B1 expression, which binds various lipoproteins like HDL for selective uptake without simultaneous conversion is not elevated 38. Subsequent tissue staining of the heart indicated that muFHR1-/-ApoE-/- mice also show less atherosclerotic plaques in the aorta or heart tissues.

Previously and herein, we demonstrate that FHR1 binds preferentially to oxLDL, as well as to oxApoB100, and that both FHR1 levels and lipid oxidation levels are high in serum from ACVD patients. Increased LDL cholesterol levels due to genetic predisposition or dietary choices, combined with lack of exercise, are major contributors to cardiovascular diseases in mice and humans and infiltration of apoB-containing LDL into the artery wall is the critical event that initiates plaque formation 6, 39, 40. Thereby native LDL is not taken up by macrophages, but modified LDL promotes foam cell formation. Modifications convert LDL into desialylated LDL which becomes more sensitive to oxidation and receptor-mediated uptake by macrophages. Desialylated LDL which is denser compared to native LDL (small LDL) 41 - 43 acts highly atherogenic.

Re-analysis of our ACVD cohort confirms the strong association of FHR1 with atherosclerosis in ACVD patients, as FHR1 levels correlate significantly with total cholesterol, clinically marker non-HDL cholesterol, and ApoB100 levels in these patients. Healthy aged control individuals already express a weak correlation of FHR1 with non-HDL cholesterol, supporting the hypothesis that FHR1 plays a crucial role in developing atherosclerosis when plasma FHR1 and oxLDL increase. That FHR proteins are associated with lipoprotein particles has been described previously. In 1996, we identified FHR4 as a lipoprotein in chylomicrons 33 and later FHR1 and FHR2 were identified as major components of lipoprotein particles suggesting for the FHR proteins a general role in lipid metabolism.

The results presented here also support data described in our previous study, which revealed a significantly lower frequency of FHR1 deficiency among ACVD patients than among healthy controls 15. As atherosclerotic processes also happen in the heart resulting in inflammatory cardiac fibrosis and reduced microvascular density, FHR1 may enhance also cardiac dysfunction in atherosclerosis. Recent studies highlight that reducing serum lipid levels effectively reverses early ventricular dysfunction and protects the heart. Similarly, reducing plasma LDL levels (e.g. via statins) or inhibiting inflammation (e.g. via canikinumab) attenuates atherosclerosis 44 - 48. LDL is also important in the context of other diseases such as cancer; indeed, recent epidemiological studies demonstrate a link between LDL and oxLDL and development of breast, colorectal, and pancreatic cancers. OxLDL can deliver cholesterol into cancer cells, thereby enhancing inflammation, cell proliferation, and metastasis 49. In summary, we show here that FHR1 (muFHR1) may play an important role in lipid homeostasis. Elevated levels of oxLDL bind FHR1 which supports inflammation and coagulation, along with all the associated secondary effects in different organs (particularly the heart), resulting in lipotoxicity. Thus, FHR1 may evolve as a new promising target in atherosclerosis. However, many open questions remain such as whether FHR1 binds to more atherogenic, desialylated LDL and how FHR1 deficiency modulates LDL and HDL concentrations in detail, elementary processes of the lipid metabolism. These questions require further investigations. The study has several limitations: The number of tested animals is very low and needs further confirmation. Also, the number of atherosclerotic patients is small relative to the high prevalence of the disease in the population and needs to be investigated in other cohorts. Whether FHR1 plays a similar role in humans as described here in mice is a topic for further investigation, as is whether inhibition of FHR1 reduces atherosclerosis in humans or whether this is compensated for by other FHR.

## Supplementary Material

Supplementary figures.

## Abbreviations

ABCG5: ATP-binding cassette sub-family G member 5
ACAT2: acetyl-CoA acetyltransferase 2
ApoA: apolipoprotein A
ApoB100: apolipoprotein B100
ACVD: atherosclerotic cardiovascular disease
BMI: body mass index
CCL2: CC-chemokine ligand 2 (MCP-1)
*CFHR1*: complement factor H-related gene 1
FHR1: complement factor H-related protein 1
*CFHR3*: complement factor H-related gene 3
CYP7A1: cytochrome P450 family 7 subfamily A member 1
EMR2: epidermal growth factor-like module-containing mucin-like hormone receptor-like 2
FHR3: complement factor H-related protein 3
HDL: high-density lipoprotein
HMGCR: 3-Hydroxy-3-methylglutaryl-CoA-Recuctase
IDL: intermediate density lipoprotein
HUVEC: human umbilical cord endothelial cell
LDL: low-density lipoprotein
M-CSF: macrophage colony-stimulating factor
oxLDL: oxidized LDL
LDLR: low density lipoprotein receptor
LPL: lipoprotein lipase
SDF-1: stromal cell-derived factor 1
SR-B1: scavenger Receptor B1
TG: triglycerides
TIMP-1: tissue inhibitor of metalloproteinase 1
VCAM1: vascular cell adhesion protein 1
VLDL: very low-density lipoprotein
