# Supplementary material for: Deletion of the Mouse Homolog of Human FHR1 (muFHR1) Alleviates Atherosclerosis in ApoE-/- mice
Source: Int J Med Sci. 2026 Jan 1;23(2):585–99. doi: 10.7150/ijms.114990 (PMC12825125; doi:10.7150/ijms.114990)
Supplement: Supplementary file 1 — Supplementary figures. [file ijmsv23p0585s1.pdf]

Deletion of the mouse homolog of human FHR1 (muFHR1) alleviates atherosclerosis in ApoE-/- mice

Luce Perie[1]*, Sarah M. Herr[2]*, Tomas Ghebreslassie[1], Sonia Wulf[3], Ina Löschmann[1], Andjela Sekulic[4], Abdulhadi Suwandi[2], Anna-Karina B. Maier[5], Luca Rowlin[5], Berit Jungnickel[6,7], Sascha Schäuble[8], Gianni Panagiotou[7,8,9], Olaf Strauß[4], Thorsten Wiech[3], Peter F. Zipfel[1,7], Svante L. H. Zipfel[10,o] and Christine Skerka[1,11,o],

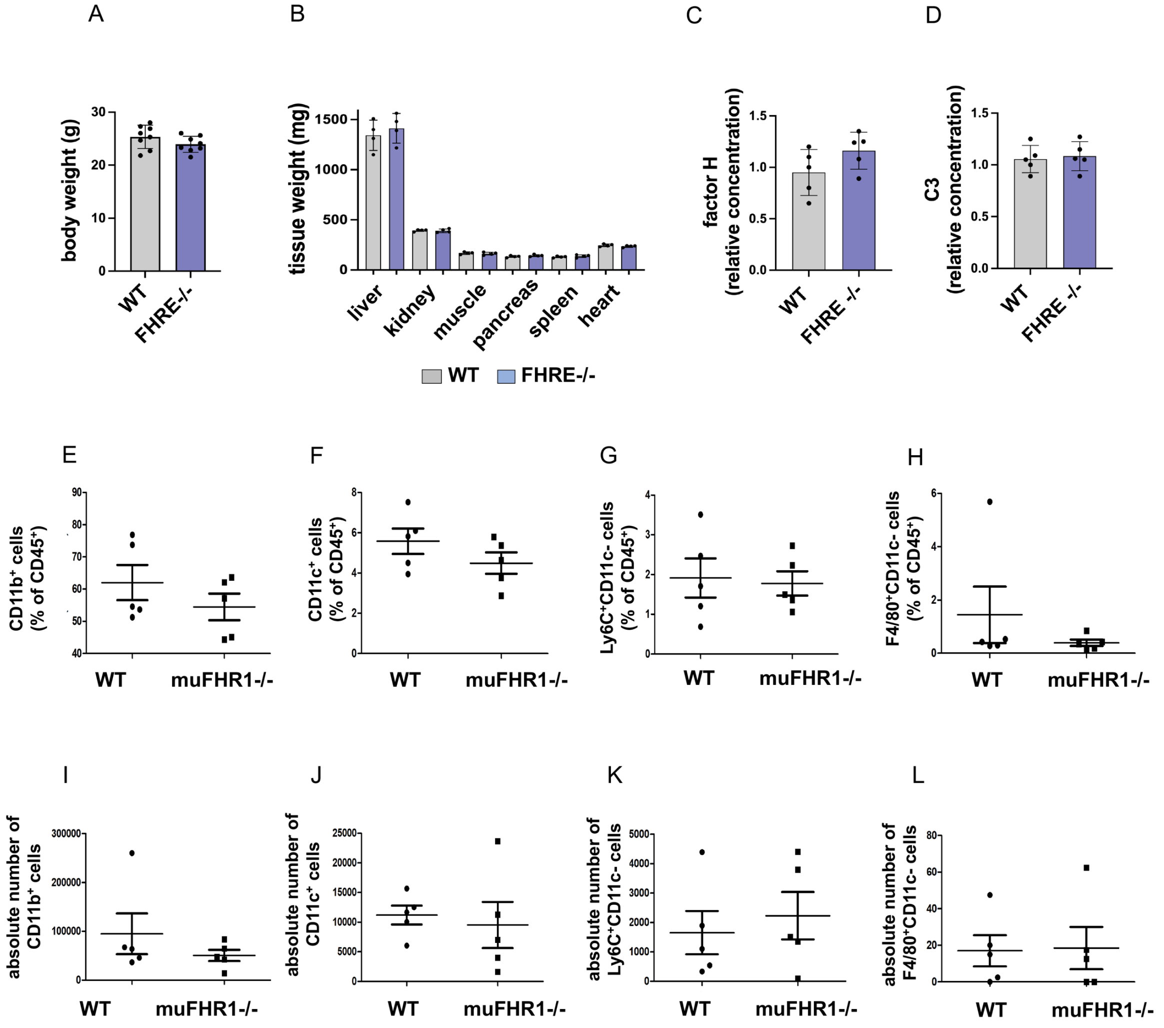

Supplementary Figure 1. Comparison between WT and muFHR1-/- mice. (**A**) The body weight of muFHR1-/- mice (n=8), as well as (**B**) the weight of various organs, were similar to that of wild-type (WT) animals (unpaired Student's t-test, n=4). (**C**) There was no difference in serum levels of factor H and (**D**) complement C3 between muFHR1-/- mice and WT mice, as measured by ELISA (unpaired Student's t-test, n=5). **(E – F)** muFHR1-/- mice show no significant differences in percentages of myeloid cells ($CD11b^+$) of CD45+ leucocytes, of dendritic cells ($CD11c^+$), of monocytes ($Ly6C^+CD11c^-$) and macrophages ($F4/80^+CD11c^-$). (**I – L**) There was also no significant difference in the absolute number of cells measured.

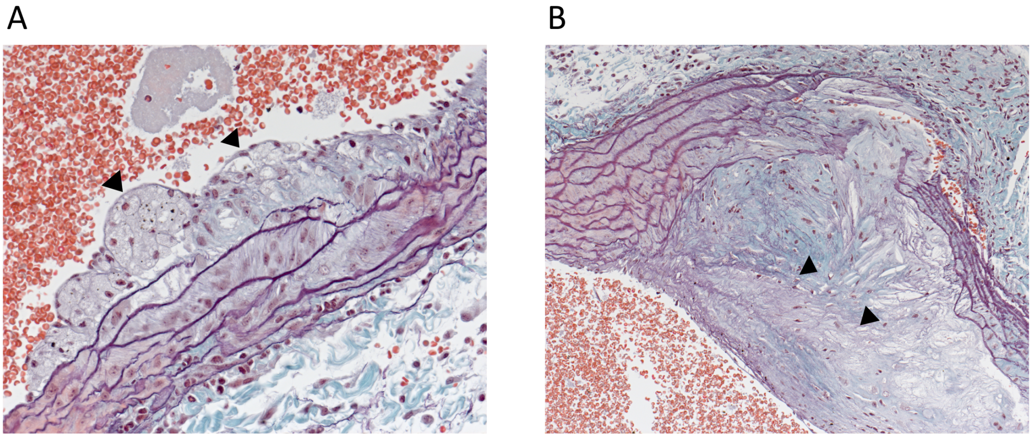

Supplementary Figure 2: The muFHR1+/+ApoE-/- mouse displays atherosclerotic deposits in the heart Cross sections of the heart of 40 weeks old muFHR1-/-ApoE-/- mouse stained with Masson-Goldner-Elastica shows (**A**) fatty deposits on the aortic wall with foam cells (triangle) and (**B**) a plaque close to the aortic valve containing cholesterol crystals (triangles, magnification x 400).

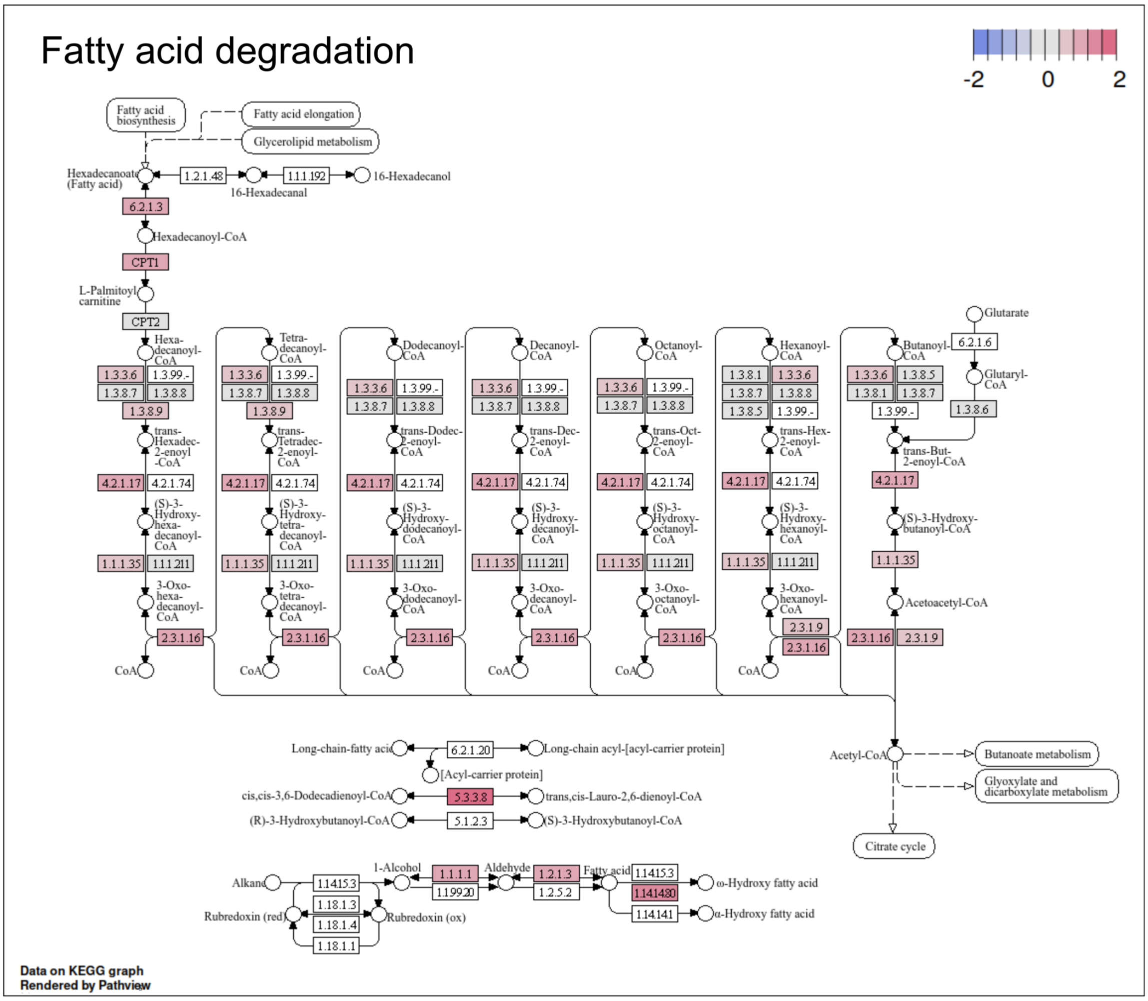

Supplementary Figure 3: Influence of muFHR1 on fatty acid degradation. muFHR1 deficiency enhances cytochrome P450 oxidoreductases Cyp3a16, Cyp2c29 and Cyp2a22 which are involved in fatty acid degradation.

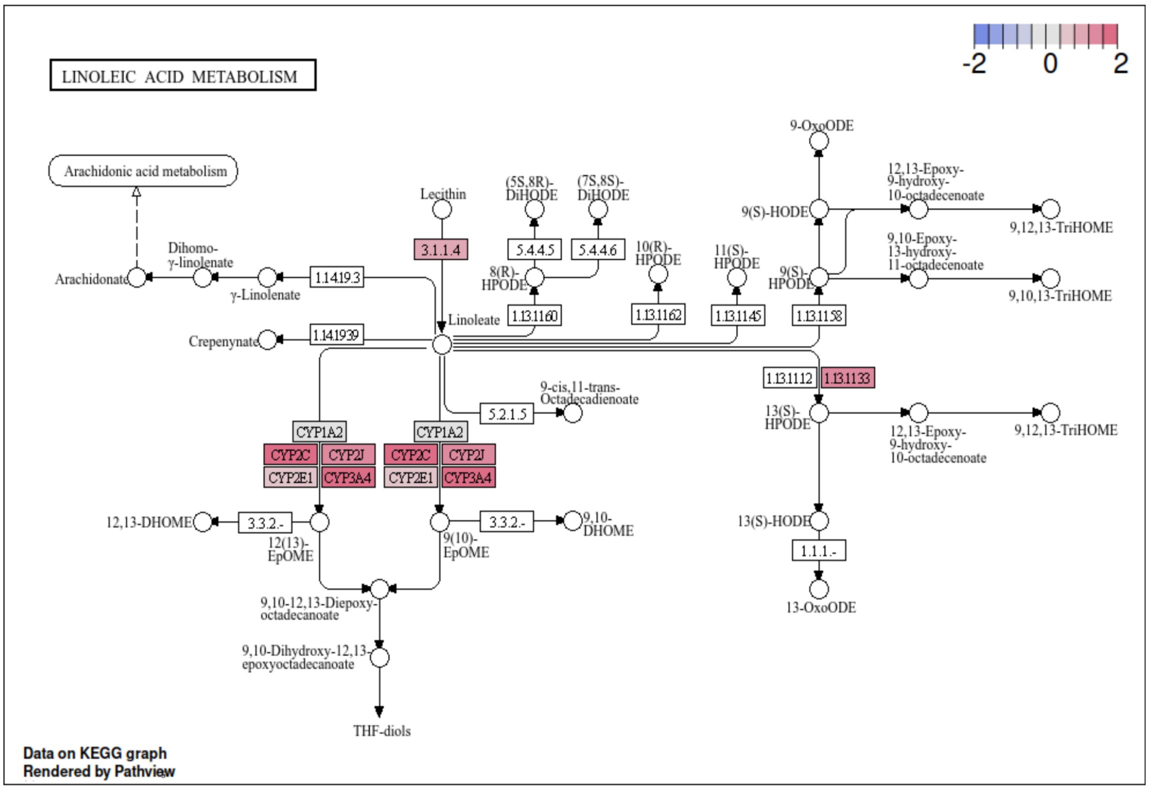

Supplementary Figure 4: linoleic acid metabolism pathways. muFHR1 deficiency enhances cytochrome P450 oxidoreductases Cyp3a16, Cyp2c29 and Cyp2a22 and enhances Linoic acid metabolism.

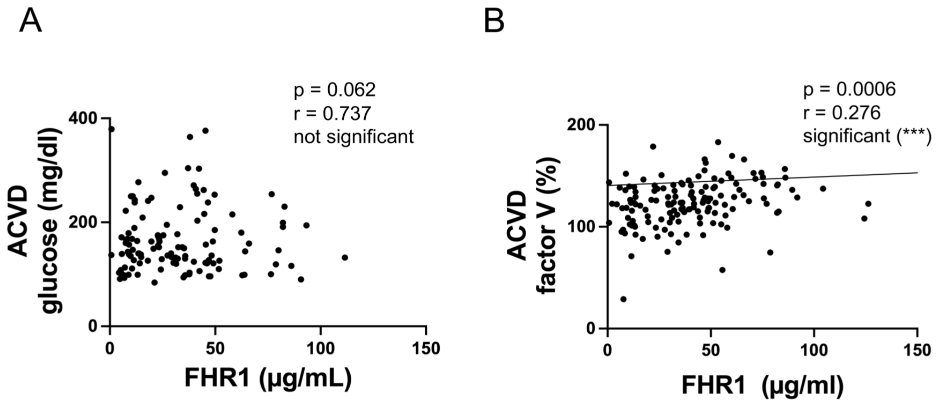

Supplementary Figure 5: FHR1 serum levels correlate with factor V concentrations but not with glucose. FHR1 serum concentrations in ACVD patients do not correlate with (**A**) glucose levels (n=117, Spearman correlation) but (**B**) highly significant with factor V (***p = 0.0006, n=151, Spearman correlation) which represents an indicator of coagulation and risk of myocardial infarction.
